# DIFFERENCES IN HOW OLDER ADULTS ARE PORTRAYED IN CHILDREN’S MEDIA

**DOI:** 10.1093/geroni/igae098.2727

**Published:** 2024-12-31

**Authors:** Hallory Domnick, Celine Manternach, Melinda Heinz

**Affiliations:** University of Northern Iowa, Cedar Falls, Iowa, United States; University of Northern Iowa, Cedar Falls, Iowa, United States; University of Northern Iowa, Cedar Falls, Iowa, United States

## Abstract

The purpose of this project was to analyze how older adults were portrayed in children’s media. Children are shaped by the media that they consume, and since the media often features negative or inaccurate portrayals of older adults, it has the potential to impact how children view this population. A total of 30 children’s picture books (published in the last 10 years) and 15 children’s shows on Disney+ and PBS Kids were analyzed for their portrayal of older adults. Each piece of media was analyzed for examples of: stereotypes/myths, positive and negative portrayals, and roles older adults engaged in. The most common stereotype/myth identified was that older adults frequently needed to use assistive devices (such as a cane) or were portrayed with exaggerated physical differences (deep wrinkles, white hair). Positive portrayals of older adults included grandparents being a source of physical affection and that spending time with grandparents was fun. Older adults were frequently portrayed as grandparents and rarely depicted in other roles. While grandparenthood may be a common role for older adults, older adults also occupy other roles and that should be better reflected in children’s media. However, some authors did include depictions of older adults identifying as LGBTQ+, demonstrating an awareness that older adults are also included in this community. Overall, older adults were more likely to be portrayed as loving and caring in children’s literature compared to television. PBS Kids compared to Disney+ was more likely to depict older adults positively as wise and knowledgeable.

